# Tigecycline Immunodetection Using Developed Group-Specific and Selective Antibodies for Drug Monitoring Purposes

**DOI:** 10.3390/bios13030343

**Published:** 2023-03-04

**Authors:** Inna A. Galvidis, Yury A. Surovoy, Sergei V. Tsarenko, Maksim A. Burkin

**Affiliations:** 1 I. Mechnikov Research Institute for Vaccines and Sera, Moscow 105064, Russia; 2 Faculty of Medicine, M.V. Lomonosov Moscow State University, Moscow 119991, Russia; 3 Federal Center for Treatment and Rehabilitation Ministry of Health, Moscow 125367, Russia

**Keywords:** tigecycline, therapeutic drug monitoring, ELISA, group and selective specificity, pharmacokinetics

## Abstract

Tigecycline (TGC), a third-generation tetracycline, is characterized by a more potent and broad antibacterial activity, and the ability to overcome different mechanisms of tetracycline resistance. TGC has proven to be of value in treatment of multidrug-resistant infections, but therapy can be complicated by multiple dangerous side effects, including direct drug toxicity. Given that, a TGC immunodetection method has been developed for therapeutic drug monitoring to improve the safety and efficacy of therapy. The developed indirect competitive ELISA utilized TGC selective antibodies and group-specific antibodies interacting with selected coating TGC conjugates. Both assay systems showed high sensitivity (IC50) of 0.23 and 1.59 ng/mL, and LOD of 0.02 and 0.05 ng/mL, respectively. Satisfactory TGC recovery from the spiked blood serum of healthy volunteers was obtained in both assays and laid in the range of 81–102%. TGC concentrations measured in sera from COVID-19 patients with secondary bacterial infections were mutually confirmed by ELISA based on the other antibody–antigen interaction and showed good agreement (R^2^ = 0.966). A TGC pharmacokinetic (PK) study conducted in three critically ill patients proved the suitability of the test to analyze the therapeutic concentrations of TGC. Significant inter-individual PK variability revealed in this limited group supports therapeutic monitoring of TGC in individual patients and application of the test for population pharmacokinetic modelling.

## 1. Introduction

Tigecycline (TGC) is the first in the class glycylcycline antibiotic approved for treatment of complicated skin and soft tissue infections, intra-abdominal infections and community-acquired pneumonia. This third-generation tetracycline is characterized by more potent activity than tetracyclines of previous generations. TGC demonstrates a wide antimicrobial spectrum which includes both gram-positive and gram-negative bacteria, and anaerobes [1]. Importantly, it is effective against tetracycline-resistant organisms with efflux and ribosomal protection mechanisms of resistance and often retains activity against carbapenem-resistant strains of *A. baumannii* and *Enterobacteriaceae*, as well as methicillin-resistant *S. aureus*, which makes it an attractive agent for management of infections caused by multidrug-resistant (MDR) microorganisms [2].

Despite excellent in vitro activity, post-marketing trials of TGC have shown an increased risk of death compared to other drugs. Indeed, based on a large meta-analysis in over seven thousand patients, TGC therapy is accompanied by an increased risk of death and treatment failure [3]. This finding is commonly attributed to the failure of target concentration achievement, and high-dose TGC therapy has been associated with increased survival [2]. However, TGC therapy could be complicated by such hazardous events as drug-induced liver failure and coagulopathy, the latter being dose-dependent [4]. Target pharmacokinetic/pharmacodynamic (PK/PD) values to improve efficacy have been proposed recently [5], whereas the thresholds for toxicity are yet to be established.

TGC pharmacokinetics in healthy volunteers has been described in multiple studies. This is a highly lipophilic drug with a large volume of distribution (7–10 L/kg) [6]. Systemic clearance is around 0.2–0.3 L/h/kg, with most of TGC excreted as the parent drug with bile. Renal excretion constitutes a minor route. TGC metabolism is minimal, the dominant metabolites, 9-aminomynocycline and glucuronide, are excreted with urine [7]. Importantly, TGC pharmacokinetics in critically ill patients is characterized by substantial inter-individual variability [8,9].

Overall, available data suggests that the introduction of TGC monitoring assays in clinical practice might improve the efficacy and safety of therapy [9,10]. Indeed, large PK studies are needed to investigate TGC toxicity and establish the therapeutic window. Furthermore, such monitoring tools will allow the TGC dosage regimens to be individualized for optimal therapy [11,12]. The aim of this study is to develop a robust assay based on immune recognition for TGC quantification in patient serum. TGC immunoassay, which is a simple, inexpensive, highly sensitive, and high-throughput alternative to physico-chemical analytical methods, has been previously reported once [13]. In the present study, we complement the previous positive attempt with an alternative design of immunogen and coating conjugates and explore the applicability of group-specific and selective antibodies for TGC monitoring purposes.

## 2. Material and Methods

### 2.1. Chemicals

European Pharmacopoeia Reference Standard of tigecycline (TGC) was used as a reference. Eravacycline (EVC, XeravaTM) was from Tetraphase Pharmaceuticals (Watertown, MA, USA), minocycline hydrochloride (MNC) was from Serva (Heidelberg, Germany), and lymecycline (LC) was from Galderma SA (La Defense, France). The other tetracyclines, namely tetracycline (TC), chlortetracycline (CTC), oxytetracycline (OTC), doxycycline (DC), and methacycline (MTC), as well as horseradish peroxidase (HRP), complete (CFA) and incomplete (IFA) Freund’s adjuvants, bovine serum albumin (BSA), transferrin (TF), formaldehyde, sodium periodate, and sodium borohydride were acquired from Chimmed (Moscow, Russia). Gelatin (GEL) was a product (#170-6537) of Bio-Rad (Hercules, CA, USA). Goat anti-rabbit IgG purchased from MP Biomedicals (Solon, OH, USA) was coupled with HRP using Nakane et al.’s method [14] to prepare GAR-HRP conjugate. The preparation and properties of group-specific anti-tetracyclines rabbit antibodies (#32, anti-BSA-TC(f)) were described earlier [15].

The coating buffer was 0.05 M carbonate-bicarbonate buffer (CBB, pH 9.6). The washing buffer and standard/sample dilution buffer were 0.15 M phosphate-buffered saline containing 0.05% Tween 20 (PBST, pH 7.2). The buffer for antibody and GAR-HRP was 1% BSA-PBST. The TMB-based substrate solution was provided by Bioservice (Moscow, Russia). Stop solution for termination of enzymatic reaction was 1 M H_2_SO_4_. High-binding polystyrene 96-well plates were from Costar (Corning, Durham, NC, USA).

### 2.2. Synthesis of Conjugated Antigens

An alternative immunogen was prepared using TGC as a hapten. For this, BSA (3 mg, 30 nmol) in 0.3 mL of water was added to 100 eqs of TGC (1.76 mg, 3 µmol) in 0.5 mL of buffers 1/0.1 M MES (pH 5.5), 2/0.15 M PBS (pH 7.4), and 3/CBB (pH 9.6). Each mixture was supplemented with 0.4 mL of formaldehyde and stirred using a magnetic stirrer for 3 days at 30 °C. Then, reaction mixtures were dialyzed against PBS 3 × 5 L for 2 days to remove excess unreacted reagents. The resultant dialysates designated as BSA-TGC(f)-5.5, BSA-TGC(f)-7.4, and BSA-TGC(f)-9.6 were stored as 1 mg/mL solutions in 50% glycerol at −20 °C until use.

The same procedure was conducted for the preparation of coating conjugates based on GEL as a carrier. During formaldehyde condensation, three temperature regimes were maintained, and the resultant conjugates were designated as GEL-TGC(f)-25C, GEL-TGC(f)-37C, and GEL-TGC(f)-50C.

Heterologous coating conjugate GEL(pi)-TGC was synthesized using periodate oxidation of GEL according to the procedure described in [16] for natamycin. Briefly, GEL (8 mg, 50 nmol) was oxidized by sodium periodate (100 eqs) in 1 mL of water for 20 min at RT. The excessive periodate was then removed by overnight dialysis against 5 L of water. One hundred molar equivalents of TGC in CBB (pH 9.6) was added to the oxidized GEL and stirred for 2 h at RT. Sodium borohydride (50 µL, 4 mg/mL) was added to reduce a Schiff base product, stirred for additional 2 h at RT, and then subjected to dialysis.

### 2.3. Antibody Preparation

The immunization procedure was carried out in accordance with the guidelines for the care and use of laboratory animals in biomedical research and was approved by the Ethical Committee for the Care and Use of Animals of I. Mechnikov Research Institute for Vaccines and Sera.

Rabbits (2.0–2.5 kg) were obtained from the Scientific and Production Centre for Biomedical Technologies (Elektrogorsk, Russia). The animals were kept for one or more weeks to adapt to vivarium conditions and then were immunized with BSA-TGC(f). Initial immunization was performed with 100 μg of the immunogen emulsified in CFA, which was injected subcutaneously at multiple points on the back. Subsequent monthly boosters were performed with a stepwise decreasing dose of the immunogen [17]. One week after each booster, blood samples were taken from the marginal ear vein of the rabbits. The resulting sera were used to control the immune response. An equal volume of glycerol was added to the sera which then were stored at −20 °C.

### 2.4. Competitive Indirect ELISA

TGC quantification was performed according to the generally accepted procedure of competitive indirect ELISA and included the following steps:

1. Coating of antigens. GEL-based conjugates at the optimum concentration in CBB were coated on the plates (100 µL per well) overnight at 4 °C.
2. Antibody binding (competitive step). Working antibody solution prepared in 1%BSA-PBST (100 µL) was added to the wells together with 100 µL of TGC standard (1000–0.01 ng/mL, and 0 ng/mL) in PBST or samples and incubated for 1 h at 25 °C.
3. Detection of bound antibody using GAR-HRP. The latter reagent in 1%BSA-PBST was added in the amount of 100 µL per well and incubated for 1 h at 37 °C. All the steps described above were completed by washing three times with PBST to remove unreacted reagents.
4. Enzymatic step. The substrate mixture (100 µL) was added to the wells, and after 0.5 h the reaction was terminated with 100 µL of the stop solution. Colored reaction product intensity was read at 450 nm using a LisaScan spectrophotometer (Erba Manheim, Czech Republic).

The optimal concentrations of antibody and coating antigens were determined in checkerboard titration experiments. Conjugates coated on the plates from solutions with different concentrations were bound by antibodies diluted to varying degrees. Pairs of immunoreagents whose binding absorbance at 450 nm was of 0.8–1.2 were then compared in a competitive assay to choose the most sensitive variant.

The inhibitory activity of each concentration of the TGC standard was expressed as B/B0—the percentage of the output signal at a certain concentration of the standard to the maximum signal at zero concentration of the standard. Four-parameter-fitted standard curves were constructed as a function ‘B/B0 vs. analyte concentration’ to determine 50% inhibition concentration as sensitivity (IC50), limit of detection (IC10), and dynamic range (IC20–IC80) [18] for a competitive assay of TGC. Cross-reactivity of antibody toward other tetracyclines was calculated as the ratio IC50 TGC/IC50 analogue.

### 2.5. Sample Collection, Pretreatment and Recovery Examination

Blood serum samples were collected from healthy volunteers and from 4 critically ill patients treated with TGC in MEDSI Clinical Hospital #1. The informed consent form was signed by the patients or their legal representatives. The study was approved by MEDSI Clinic Independent Ethical Committee, Moscow, Russia (Protocol #29 15 April 2021).

The assay was used to quantify TGC serum concentration in adult patients (#1–#4) with COVID-19 and secondary bacterial infections caused by MDR strains of *K. pneumoniae*, *E. faecium*, *A. baumannii*, and *K. pneumoniae*, respectively. TGC was used at a dose 50–100 mg every 12 h in combination with meropenem or polymyxin B at the discretion of the treating physician. Patients received a loading dose twice the maintenance dose on the first day of therapy. For PK analysis, 6–8 blood samples were collected over 12 h in each patient (n = 3) after at least 48 h of TGC therapy: before the infusion; 5 min, 30 min, 1 h, 2 h, 4 h, 8 h, and 11 h after the infusion. 

Each sample was collected in EDTA K4 tubes, centrifuged at 4000 rpm for 10 min, after which supernatant was withdrawn, frozen at −20 °C, and analyzed within a month.

Before the analysis, serum samples were thawed and treated with TCA for deproteinization according to the previously described procedure [19]. The precipitated protein was separated by centrifugation for 5 min at 6800× *g*, and the resulting supernatant appropriately diluted with PBST was tested by ELISA.

The blank sera from healthy volunteers were spiked with TGC standard at 0.3 and 1.5 mg/L, incubated 1 h at 37 °C, treated as above, and analyzed by ELISA to determine the rate of recovery as a percentage between TGCfound and TGCspiked.

GraphPad Prism 8.0 software served to generate standard curves and calculate the concentration of TGC in the samples. PKanalix version 2020R1 (Lixoft SAS, Antony, France) was used for non-compartmental PK analysis.

## 3. Results and Discussion

### 3.1. Preparation and Characteristics of Conjugated Antigens

**Figure 1:**
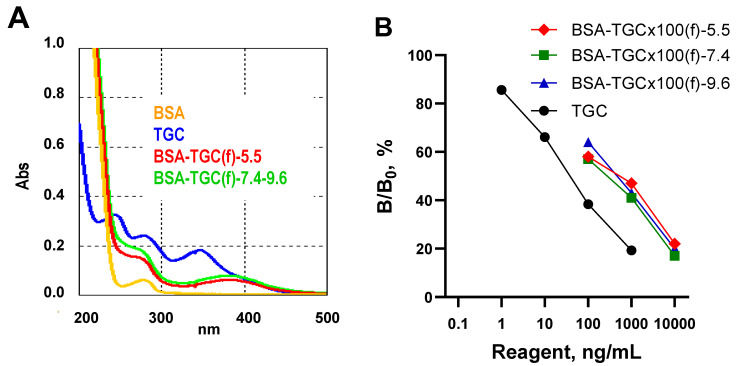
(**A**) The UV spectra of protein carrier BSA, hapten TGC, and resultant conjugate BSA-TGC(f). BSA and conjugate concentrations were 0.1 mg/mL, and TGC concentration was 0.01 mg/mL in water. (**B**) Inhibitory activity of prepared conjugates in ELISA using #32 antibody and TF(pi)-CTC as a coating antigen [15].

In a recent work by Xu et al. [13], to obtain antibodies against TGC, its closest analogue, 9-amino-minocycline, was used as an immunizing hapten, and its conjugation was carried out by the diazonium and glutaraldehyde methods. Here we attempted to retain the distinctive structural feature of TGC, the 2-(tert-butylamino)acetamide moiety, and used the entire TGC molecule as a hapten. Within this approach, one of the effective conjugation methods for tetracyclines, the Mannich reaction [15], was applied to prepare TGC-based conjugates. The UV spectra of conjugates demonstrated the changes occurred in the spectra of BSA-TGC(f) compared with BSA spectrum. Thus, as a result of conjugation, the spectral characteristics of TGC (two peaks at 243 nm and 278 nm) and BSA (peak at 278 nm) fused into a smoothed shoulder on the spectral curve at about 260 nm. The third most pronounced TGC peak at 347 nm in the conjugate was shifted to 383 nm (Figure 1A). The spectra of BSA-TGC(f)-7.4 and BSA-TGC(f)-9.6 did not differ from each other but were somewhat more intense compared to BSA-TGC(f)-5.5. Therefore, the conjugation of tetracyclines using Mannich condensation under neutral and alkaline pH conditions was preferable, in contrast to the recommendations in [20]. The inhibitory reactivity of conjugates in competitive ELISA may reflect the number of reactive epitopes on the tested conjugates [21] and hence their specific immunogenic activity (Figure 1B). Thus, BSA-TGC(f) conjugates prepared at different pH were compared for their inhibitory activity. BSA-TGC(f)-7.4 turned out to be, although slightly, more active than other conjugates, which is in full agreement with the spectral data. Therefore, BSA-TGC(f)-7.4 was chosen as an immunogen. The study of GEL-TGC(f)-25C, GEL-TGC(f)-37C, and GEL-TGC(f)-50C spectra showed a slightly more efficient conjugation in the latter case.

The formation of heterologous conjugate GEL(pi)-TGC was confirmed immuno-chemically by antibody binding when it was used as a coating antigen.

### 3.2. Antibody Preparation, ELISA Development and Characteristics

In addition to the previously developed antibodies with group recognition of tetracyclines #32 [15], the present research was aimed to obtain a new antibody to TGC for comparison of their analytical properties.

**Figure 2:**
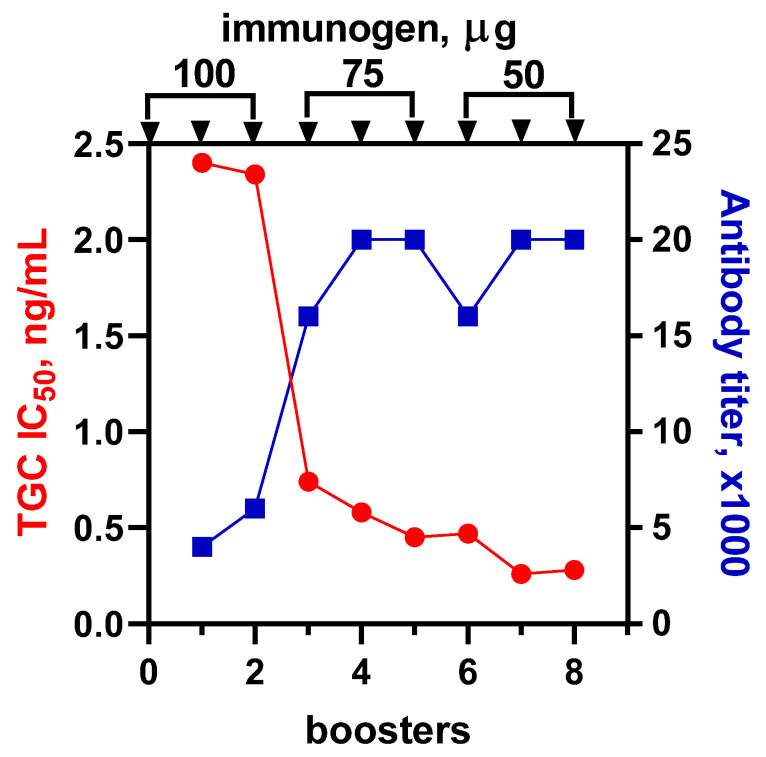
Sensitivity and working titer of anti-BSA-TGC(f) in the dynamics of immunization. Each titer value is represented by an average (n = 3) working dilution of the antibody, providing binding absorbance in the range of 0.8–1.2. IC50 values were calculated using the standard curves derived from corresponding antibodies.

Immunization was carried out with a stepwise decreasing dose of BSA-TGC(f) according to the scheme (3 × 100–3 × 75–3 × 50 µg) shown in Figure 2. The rise of the working antibody titer ceased with the fifth injection of the immunogen, reaching 20,000. However, a more important criterion, the antibody maturation, gradually improved and reached its best value after the seventh booster immunization. The sensitivity (IC50) of the assay based on #100/7 antibody was 0.23 ng/mL. These antibodies were chosen for further experiments.

Both anti-BSA-TC(f) (Ab#32) and anti-BSA-TGC(f) (Ab#100) antibodies were tested for their reactivity with two types of coating antigens, GEL-TGC(f) and GEL(pi)-TGC, to generate optimized ELISA variants. The best characteristics were found for heterologous pair of reagents [22]. The first one was based on Ab#32 and heterologous hapten coating conjugate GEL-TGC(f), whereas the second pair of reagents included Ab#100 and heterologous conjugation coating antigen GEL(pi)-TGC.

Specificity studies confirmed that the first ELISA was group-specific (Figure S1) and capable of detecting, along with first- and second-generation tetracyclines, the representatives of the third generation: TGC and EVC (Table 1). The assay based on new antibodies was TGC-selective without any cross-reactions with analogues. This serves as an argument in favor of the unique 2-(tert-butylamino)acetamide fragment of TGC being the target epitope for Ab#100 (Figure S2).

**Figure 3:**
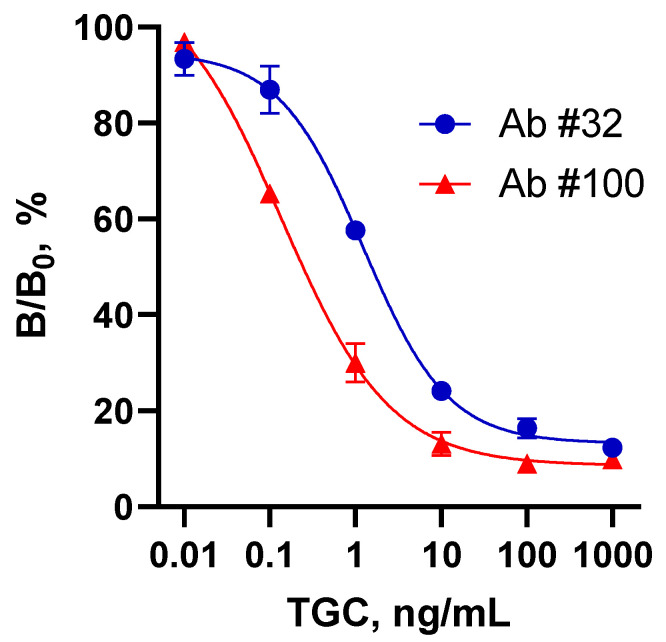
Standard curves for TGC determination in group-specific (Ab#32) and TGC-selective (Ab#100) antibody-based ELISAs. Each point is represented by an average value (n = 3) ± standard deviation.

Thus, two ELISA systems were created for TGC determination. Due to their different specificities, it can be postulated that they recognized different fragments (common and individual) of the TGC molecule; both were further employed for therapeutic drug monitoring purposes. The standard curves for these assays are shown in Figure 3, and analytical characteristics are compared in Table 2.

If the Ab#32-ELISA provided a level of sensitivity comparable to the previous report [13], then the Ab#100-based assay had an order-of-magnitude better sensitivity and strict TGC selectivity. To the best of the authors’ knowledge, no other immunoassay systems for TGC are available in the literature.

### 3.3. Sample Pretreatment and Recovery Experiments

The sensitivity of the developed tests was high enough to analyze microvolumes of biosamples after a high degree of dilution, which eliminated possible interference from their matrix. However, the deproteinization as a pretreatment procedure ensures unification of the samples, excluding possible interferences and interactions with plasma proteins [17,23], concentrations of which could vary significantly, e.g., in critically ill patients. Chromatographic methods for TGC detection, such as HPLC and LC-MS/MS, usually involve protein precipitation of serum samples with acetonitrile [24,25]. In comparison to the commonly used acetonitrile, another deproteinizing agent, TCA, has demonstrated a distinct advantage in tetracycline bioanalysis, which is its stabilization from epimerization and other degradation pathways [26]. For this reason, we have compared several sample preparation approaches involving simple dilution with assay buffer and deproteinization with TCA, MeOH, and ACN (Table 3). The obtained TGC recovery data from spiked health volunteers’ sera were satisfactory for the dilution approach (81–102%) and TCA-mediated deproteinization (74–89%) in both assay systems, whereas MeOH and ACN pretreatment of sera samples provided mostly poor recovery (24–69%). 

Comparison of PBST dilution with TCA deproteinization as pretreatment procedures performed with real serum samples did not reveal significant differences between the data. Similar results were obtained in group-specific and TGC-selective analytical systems (Figure S3). Both of the studied sample pretreatment approaches were found to be acceptable, but regardless of this, further experiments were performed with deproteinized samples.

**Figure 4:**
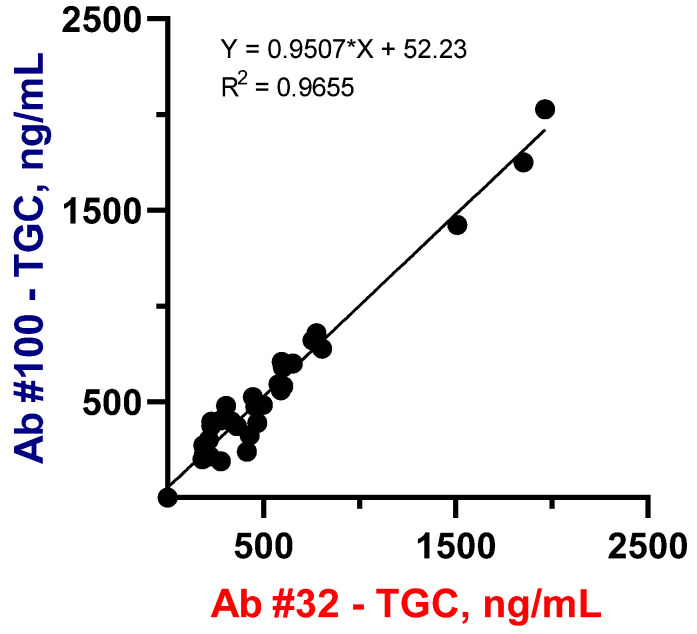
Correlation between TGC serum concentrations (n = 31) from 4 patients with sepsis obtained using two assay systems: TC group-specific ELISA (Ab#32) and TGC-selective ELISA (Ab#100). Standard errors for slope and Y-intercept were 0.03336 and 23.67, respectively.

The sera samples (n = 31) from four critically ill patients were taken to quantify TGC and establish a correlation between two assay systems. As can be seen from Figure 4, the TGC-specific (Ab#100) ELISA data were in good agreement with those obtained in the group specific (Ab#32) assay system. It can be assumed that slightly higher values in Ab#32-ELISA compared to Ab#100-ELISA (95%) could be caused by the recognition of TGC metabolites by group-specific antibodies.

The strong correlation between these two groups of data with a coefficient of 0.95 and R^2^ = 97% can serve as a mutual confirmation of the reliability of the results obtained. This data consistency also confirms the suitability of the group-specific assay system (Ab#32-ELISA) to correctly measure both TGC and other tetracycline analytes using a correspondent calibration. However, further experiments on TGC pharmacokinetics were performed using a specific tool (Ab#100-ELISA).

### 3.4. Tigecycline Pharmacokinetics in Critically Ill Patients Using the Developed ELISA

**Figure 5:**
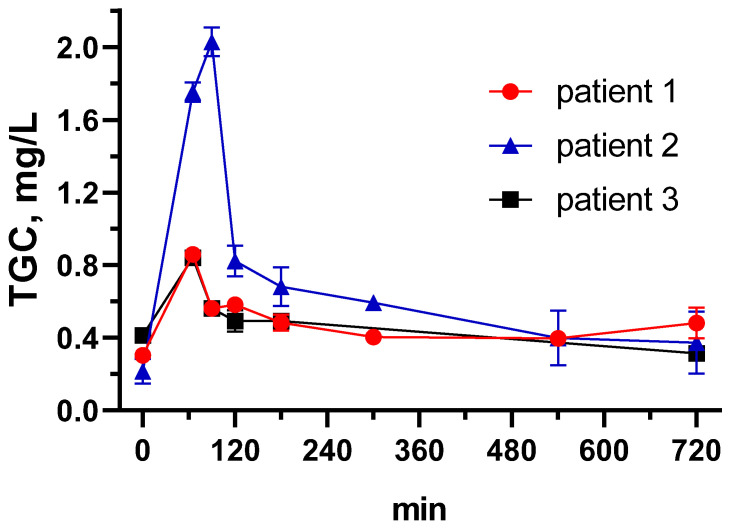
Tigecycline pharmacokinetics in critically ill patients. Each point is the average from triplicate and error bars represented by standard deviation.

Steady-state TGC pharmacokinetics was described for three out of four critically-ill patients with COVID-19 and secondary bacterial infections. Two patients received continuous venovenous hemodialysis (Patients 1 and 2); one patient was on extracorporeal membrane oxygenation (Patient 3). TGC was used as a 1 h infusion at a dose of 50–100 mg selected by the treating physician. TGC concentration curves over time are presented in Figure 5, observed PK parameters are presented in Table 4. At this point, the observed distinctions between PK parameters cannot be ascribed to any specific clinical and demographic parameter, but rather represent inter-individual PK variability in critically ill patients, as also shown in other studies [8,12]. The figure nicely demonstrates how similar serum concentrations are achieved with different doses due to individual changes of clearance and volume of distribution. Overall, the developed assay has demonstrated its suitability for the measurement of TGC therapeutic concentrations in the serum of critically ill patients, and thus may be used for population PK studies and therapeutic drug monitoring in individual patients.

## 4. Conclusions

Two immunodetection systems for TGC were developed based on indirect competitive ELISA using class-specific and TGC-selective antibodies. Using a specially selected coating antigen, a previously developed group recognition antibody was adapted to detect, in addition to the first- and second-generation tetracyclines, also the third generation representatives, TGC and EVC. The de novo-generated antibody against BSA-TGC conjugate demonstrated strict TGC selectivity. Thus, two immunoassays capable of recognizing different TGC moieties were compared as tools for therapeutic monitoring purposes. Both assays demonstrated high sensitivity with half-inhibition TGC concentrations of 0.23 and 1.59 ng/mL and satisfactory recovery from spiked volunteers’ sera of 74–102%. Sample pretreatment, such as simple dilution with assay buffer or TCA-mediated deproteinization, was both adequate and took no more than 5 min. The developed ELISAs were applied to describe TGC pharmacokinetics for three critically ill COVID-19 patients with secondary bacterial infections. Despite the limited study group of patients, the resulting PK profile varied significantly, indicating the relevance of individual therapeutic TGC monitoring. The concentrations measured by both analytical systems, differing in specificity, strongly correlated with a coefficient of 0.95 and R^2^ = 97%, confirming the validity of the data and the reliability of the tests.

## Figures and Tables

**Table 1:** Structural formula of tetracyclines and cross-reactivity (CR) profile of anti-BSA-TC(f) and anti-BSA-TGC(f).

Analyte		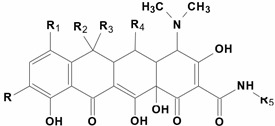						
	R	R_1_		R_2_		R_3_	R_4_	R_5_
TC EVC TGC CTC LC MNC DC OTC MTC	H NHCOCH_2_N(CH_2_)_4_ NHCOCH_2_NHC(CH_3_)_3_ H H H H H H	H F N(CH_3_)_2_ Cl H N(CH_3_)_2_ H H H		OH H OH OH OH H OH H	CH_2_	CH_3_ H CH_3_ CH_3_ CH_3_ H CH_3_ CH_3_	H H H H H H H OH H	H H H H Me-N-Lys H H H H
	**Anti-BSA-TC(f) Ab #32**					**anti-BSA-TGC(f) Ab #100**		
**Analyte**	**IC50, ng/mL**		**CR, %**			**IC50, ng/mL**		**CR, %**
TC	1.25		126.5			>1000		<0.02
EVC	1.33		119.3			>1000		<0.02
TGC	1.59		100			0.23		100
CTC	1.90		83.5			>1000		<0.02
LC	5.58		28.4			>1000		<0.02
MNC	9.08		17.5			>1000		<0.02
DC	13.08		12.1			>1000		<0.02
OTC	27.05		5.9			>1000		<0.02
MTC	62.11		2.6			>1000		<0.02

Coating antigens were GEL-TGC(f) for Ab#32 and GEL(pi)-TGC for Ab#100.

**Table 2:** Comparative analytical characteristics of immunoassays for TGC.

Assay	IC50	IC20–IC80	IC90 (LOD)	Reference
ELISA LFIA ELISA-Ab#32	2.30 nd 1.59	nd nd 0.22–18.9	0.22 15.03 0.05	[13] [13] Present work
ELISA-Ab#100	0.23	0.04–2.83	0.02	Present work

Parameters were not determined (nd) in the work.

**Table 3:** Comparison of serum samples pretreatment approaches in recovery (RC) experiments.

Sample Pretreatment	TGC Spike Level, ng/mL	Ab #32		Ab #100	
		RC, %	RSD, %	RC, %	RSD, %
PBST	1500	81.4	8.6	102.0	9.4
	300	98.1	3.4	90.8	11.5
TCA	1500	82.0	6.0	88.7	2.2
	300	73.6	9.7	80.1	4.1
MeOH	1500	24.1	16.1	25.9	15.5
	300	28.5	7.1	24.7	22.7
ACN	1500	35.8	24.3	35.1	14.7
	300	34.8	38.8	69.3	1.2

**Table 4:** Tigecycline pharmacokinetics in critically ill patients.

	Patient 1	Patient 2	Patient 3
Dose, mg/12 h	100	100	50
AUC0-24 h, mg*h/L	11.08	15.38	10.60
Cmax, mg/L	0.86	2.03	0.84
CL, L/h	18.04	13.01	9.43
Vss, L	975.49	149.96	189.56
Causative microorganism	*K. pneumoniae*	*E. faecium*	*A. baumannii*

AUC0-24 h—area under the concentration-time curve from 0 to 24 h, Cmax—maximal plasma concentration, CL—clearance, Vss—volume of distribution at steady state.

## Data Availability

The datasets used and/or analyzed during the current study are available from the corresponding author on request.

## Supplementary Materials

The following supporting information can be downloaded at: https://www.mdpi.com/article/10.3390/bios13030343/s1.
