# Supplementary material for: Tigecycline Immunodetection Using Developed Group-Specific and Selective Antibodies for Drug Monitoring Purposes
Source: Biosensors (Basel). 2023 Mar 4;13(3):343. doi: 10.3390/bios13030343 (PMC10046529; doi:10.3390/bios13030343)
Supplement: Supplementary file 1 [file biosensors-13-00343-s001.zip › biosensors-2199758-supplementary.pdf]

Tigecycline immunodetection using developed group-specific and selective antibodies for drug monitoring purposes

Inna A. Galvidis [1], Yury A. Surovoy [1,2], Sergei V. Tsarenko [2,3] and Maksim A. Burkin [1*]

[1] I. Mechnikov Research Institute for Vaccines and Sera, Moscow 105064, Russia;
[2] Faculty of Medicine, M.V. Lomonosov Moscow State University, Moscow 119991, Russia;
[3] Federal Center for Treatment and Rehabilitation Ministry of health, Moscow 125367, Russia;
* Correspondence: burma68@yandex.ru; Tel./Fax: +7 495 9172753.

Results and Discussion

Cross-reactivity examination

The specificity of the developed Ab#32-based and Ab#100-based ELISAs for TGC was judged by the inhibitory activity of related tetracyclines. The presented data demonstrate that the first assay is group-specific and allows the detection of a number of tetracyclines. (Figure S1). Whereas the assay based on new antibodies was TGC-selective without any cross-reactions with other tetracyclines (Figure S2). Cross-reactivity (CR) values calcu-lated as the ratio IC_{50} TGC/IC_{50} analogue are shown in Table S1.

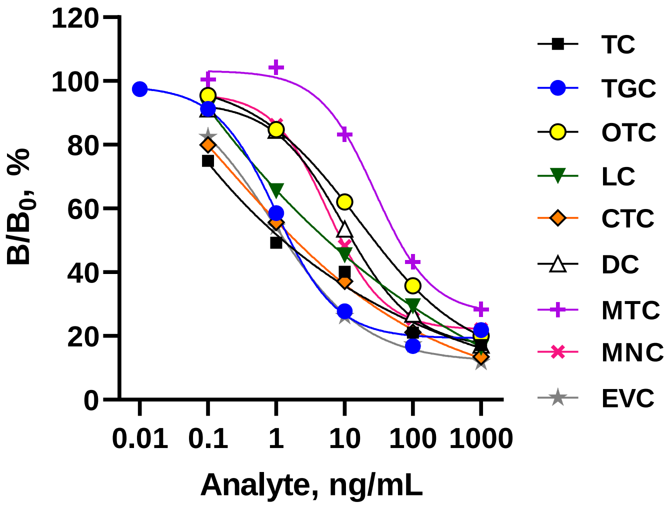

Figure S1. Standard curves of tetracyclines in indirect competitive ELISA using anti-BSA-TC(f) (Ab#32) and GEL-TGC(f) as coating antigen. Tetracycline (TC), tigecycline (TGC), oxytetracycline (OTC), lymecycline (LC), chlortetracycline (CTC), doxycycline (DC), methacycline (MTC), minocy-cline (MNC), and eravacycline (EVC).

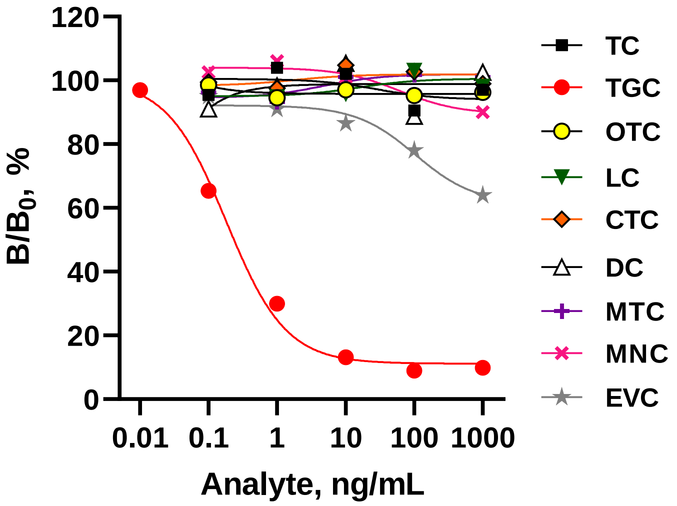

Figure S2. Standard curves of tetracyclines in indirect competitive ELISA using anti-BSA-TGC(f) (Ab#100) antibodies and GEL(pi)-TGC as coating antigen.

Table S1. Cross-reactivity (CR) profile of anti-BSA-TC(f) and anti-BSA-TGC(f).

Analyte	Ab #32		Ab #100	
	IC_{50}, ng/mL	CR, %	IC_{50}, ng/mL	CR, %
TC	1.25	126.5	>1000	<0.02
EVC	1.33	119.3	>1000	<0.02
TGC	1.59	100	0.23	100
CTC	1.90	83.5	>1000	<0.02
LC	5.58	28.4	>1000	<0.02
MNC	9.08	17.5	>1000	<0.02
DC	13.08	12.1	>1000	<0.02
OTC	27.05	5.9	>1000	<0.02
MTC	62.11	2.6	>1000	<0.02

Coating antigens were GEL-TGC(f) for Ab#32 and GEL(pi)-TGC for Ab#100.

Comparison of PBST dilution and TCA deproteinization approaches for sample pretreatment

To determine which procedure is preferable, a comparison was made between PBST dilution and TCA deproteinization for pretreatment of real serum samples. The residual matrix effect of serum proteins, whose composition can vary greatly in samples from critically ill patients, can be eliminated by TCA deproteinization and the samples will be unified. On the other hand, non-physiological conditions of acid treatment of samples can affect the analyte and require additional, albeit insignificant, time costs (5-10 min). Comparative study of 31 serum samples performed using both assays did not reveal significant differences with both assay systems to conclude either approach is preferable (Figure S3).

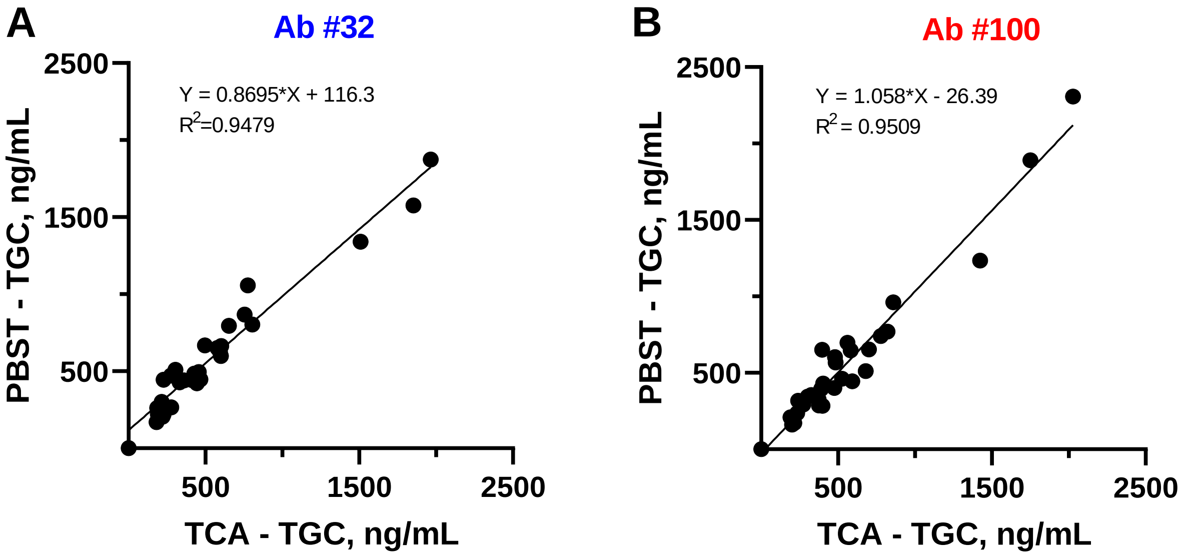

Figure S3. Correlation between sera dilution and TCA deproteinization as pretreatment approaches in TC group-specific ELISA (**A**) and TGC-selective ELISA (**B**).
